# The Application of a Foliar Spray Containing *Methylobacterium symbioticum* Had a Limited Effect on Crop Yield and Nitrogen Recovery in Field and Pot-Grown Maize

**DOI:** 10.3390/plants13202909

**Published:** 2024-10-17

**Authors:** Manuel Ângelo Rodrigues, Carlos Manuel Correia, Margarida Arrobas

**Affiliations:** 1 Centro de Investigação de Montanha (CIMO), Instituto Politécnico de Bragança, Campus de Santa Apolónia, 5300-253 Bragança, Portugal; marrobas@ipb.pt; 2 Laboratório para a Sustentabilidade e Tecnologia em Regiões de Montanha, Instituto Politécnico de Bragança, Campus de Santa Apolónia, 5300-253 Bragança, Portugal; 3 Centre for the Research and Technology of Agro-Environmental and Biological Sciences (CITAB), University of Trás-os-Montes and Alto Douro, 5001-801 Vila Real, Portugal; ccorreia@utad.pt

**Keywords:** *Zea mays*, biological nitrogen fixation, plant biostimulant, microbial inoculant, beneficial microorganisms

## Abstract

In this study, the effectiveness of an inoculant containing a nitrogen (N)-fixing microorganism (*Methylobacterium symbioticum*) was evaluated on maize (*Zea mays* L.) grown both in the field (silage maize) and in pots over two years (2021 and 2022). The field trial included the following two treatments: with (Yes) and without (No) the inoculant. The pot experiment was designed as a factorial arrangement with two factors: the application of the inoculant (Yes and No) and N applied to the soil (0, 0.4, 0.8, and 1.6 g pot^−1^). In the field, total dry matter yield (DMY) did not differ significantly between treatments, although the average DMY was higher in the inoculant treatment. In pots, the total DMY varied significantly across all N rates but was only significantly affected by the inoculant application in 2022. N fixation estimates in the field were 58.8 and 14.5 kg ha^−1^ for 2021 and 2022, respectively, representing 23.7% and 9.1% of the N recovered in the aboveground plant parts. In pots, the estimated fixed N values were −49.2 and 199.2 mg pot^−1^ in 2021 and 2022, respectively, which corresponded to −5.2% and 18.5% of the N found in the aboveground plant parts. Considering the average values obtained across the four cultivation conditions, there was a positive outcome for the treated plants. However, these values cannot be considered significant when compared to nitrogen removal in maize crops. A commercial product should provide an unequivocal and quantitatively relevant contribution to plant nutrition, which did not appear to be the case. Thus, for this inoculant to provide reliable guarantees of positive outcomes for farmers and become a useful tool in promoting more sustainable agriculture, further studies appear necessary. These studies should aim to determine in which crops and under what cultivation conditions the application of the inoculant is truly effective in enhancing N fixation and improving crop productivity.

## 1. Introduction

Maize is one of the most important crops in the world, rivaling wheat and rice. In 2022, the area under maize cultivation was ~203 M ha and grain production was ~1.16 billion metric tons [1]. Although it has less importance as a food crop compared to wheat and rice, maize is a more versatile multi-purpose crop, making it the second most widely grown crop in the world after wheat, the latter grown on ~219 M ha [1]. In the developed economies, maize grain is primarily used as a livestock feed crop with a varied role as an industrial and energy crop, with emphasis on the production of bioethanol [2]. In addition, the maize plant is also used as green forage and for silage. Maize cultivated for forage accounts for an additional 16.8 M ha annually [3,4].

Agricultural systems in which maize is grown in the world can be very diverse and with very different levels of farming intensification. Maize grown in particularly high input systems is prevalent in the Global North, where it can generate environmental externalities including land degradation, water eutrophication, and greenhouse gas emissions into the atmosphere [2]. In high-input agricultural systems, maize is one of the crops that receives the highest rates of N fertilizers [5,6,7]. Excessive use of N in agricultural fields leads to the loss of large amounts of nitrates to waterbodies, resulting in harmful algal blooms [8,9]. Irrigated crops are also responsible for the emission of N oxides into the atmosphere, because the wetting and drying cycles create favorable conditions for nitrification which is followed by denitrification [10,11]. When maize is integrated into livestock farming, it receives high amounts of farmyard manure, which tend to increase environmental problems. In addition to the loss of nitrates to watercourses and the emission of greenhouse gases into the atmosphere, the use of organic amendments increases the potential loss of dissolved organic carbon (C) [12,13] with an impact potentially harmful to aquatic ecosystems and drinking water quality [14,15]. Furthermore, animal manure is rich in phosphorus (P), especially that of non-ruminants which lack phytases [16,17]. The application of large amounts of animal manure can reduce the P retention capacity of soils, increasing the labile P fractions and exhausting P retention sites with nutrient loss to watercourses [18,19], where it exacerbates the effect of N, increasing the risks of eutrophication, hypoxia, and loss of species diversity [20].

Some crops can access high amounts of N from the atmosphere, thus needing to receive smaller amounts of N fertilizers, which makes agricultural practices more sustainable, with less energy consumption and less environmental impact. Nodulated legumes, for instance, establish symbiotic relationships with N-fixing microorganisms, commonly known as diazotrophs, accessing N that may be sufficient to meet their nutritional needs [21,22,23]. In rice (*Oryza sativa* L.) fields, the development of the aquatic fern Azolla has been promoted, since it establishes a symbiotic relationship with the N-fixing cyanobacterium *Anabaena azollae*, the fern being cultivated as green manure before rice transplantation or as an intercrop with rice [24,25], which can provide more than half of the N required for the rice crop [21,23,24]. It has also been shown that the tissues of some plants are invaded by endophytic microorganisms capable of fixing N. In the case of sugarcane (*Saccharum officinarum* L.), these microorganisms can provide the crop with more than half of its N needs [26,27]. Maize is often grown in intercropping or in rotation with legumes to reduce N fertilizer needs [28,29,30]. However, it has been demonstrated that maize can directly access atmospheric N by its own means. Some N-fixing microorganisms, such as *Herbaspirillum seropedicae*, are considered true endophytic diazotrophs predominantly associated with tropical grasses [31,32]. *H. seropedicae* can invade the roots, stems, and leaves of the host plant, mainly the apoplastic compartments [32,33]. Alves et al. [34] applied the endophytic diazotroph *H. seropedicae* strain ZAE94 to maize under field conditions. The authors found that application of the *H. seropedicae* inoculant increased the amount of N in plants owing to biological N fixation. However, the agronomic significance of bioinoculants with *H. seropedicae* for N nutrition under field conditions remains a matter of debate and improvement and is currently not comparable to the effects of rhizobia inoculation in legumes [32,34].

*Methylobacterium symbioticum* is a diazotroph recently isolated from spores of *Glomus iranicum* var. *tenuihypharum* [35]. The genus *Methylobacterium* is ubiquitous in nature, with numerous species thriving in diverse habitats [36,37]. Recently, an inoculant containing *M. symbioticum* (strain SB0023/3 T) appeared on the market for application in foliar spraying with the promise of being effective in non-legume species. The microorganism lodges in the phyllosphere where it has access to organic compounds, providing N to the plants in return [35,38]. It is well known that certain species of the genus *Methylobacterium* can live on the phyllosphere, utilizing methanol as a source of C and energy [39,40,41]. Plants release substantial amounts of methanol through their stomata as a byproduct of pectin metabolism during cell wall synthesis [42]. In this way, plants can support bacterial activity and enhance N fixation capacity. A previous study demonstrated that the presence of the strain SB0023/3 T reduces the activity of nitrate reductase, which is evidence of an increased presence of ammonium N, an intermediate compound in N fixation [38]. Additionally, the microorganism promotes the production of phytohormones that stimulate plant growth, thereby increasing plant productivity [35]. Thus, given the high N rates usually used in maize, some contribution of N via biological fixation would have enormous ecological significance.

To better understand the ability of this microorganism to fix N, two experiments were conducted, one in the field and the other in pots. In the field, only two treatments were used, with and without application of the inoculant to maize fertilized with 70% of the N recommended by the laboratory, as suggested by the manufacturer. In pots, a factorial experiment was established with and without the application of inoculant and four rates of mineral N (equivalent to 0, 40, 80, and 160 kg ha^−1^ of N). The hypothesis raised for the field and pot experiments was whether the inoculant improves crop productivity and increases the amount of N recovered by maize. Based on the pot experiment, a second hypothesis was raised as to whether the nutritional status of the plants, created by different rates of N fertilizer, affects the performance of *M. symbioticum* in its ability to fix N.

## 2. Materials and Methods

### 2.1. Experimental Conditions

**Figure 1:**
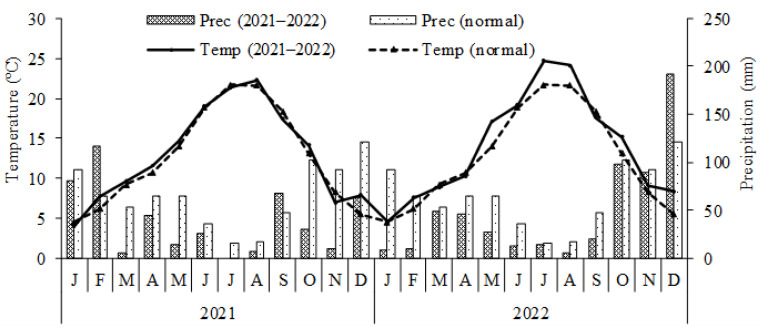
Monthly average temperature and accumulated precipitation of the climatological normal and recorded during the experimental period at the meteorological station of Quinta de Santa Apolónia, Bragança.

This study involved a field trial and a pot experiment, both carried out in Bragança, Northeastern Portugal, during two consecutive growing seasons, in 2021 and 2022. The region benefits from a Mediterranean warm summer climate (Csb), according to the Köppen–Geiger classification. The average annual temperature is 12.6 °C and the total annual precipitation is 772.7 mm [43]. The records of average air temperatures and monthly precipitation for the experimental period are shown in Figure 1.

The field trial was carried out on a plot subjected to an eight-year crop rotation, where four years of forage-maize are followed by four years of temporary pasture. The experiment took place in the third and fourth years of the maize phase. The soil is a eutric Fluvisol [44], developed in a fluvial deposit, with a sandy loam texture. In the year of installation of the experiment, the soil of the plot presented the properties shown in Table 1, determined from composite samples taken at a depth of 0–0.20 m. In the pot experiment, soil from the 0–0.20 m layer was used, taken from a plot that had remained uncultivated the previous year. The soil is a Regosol of colluvial origin [44], with a sandy clay loam texture. Other properties of the soils used in this study are shown in Table 1.

### 2.2. Experimental Designs

The field trial was carried out with only two treatments, with and without application of the inoculant containing *M. symbioticum*. The experimental plot received 70% of the amount of N estimated as necessary for the maize crop, as recommended by the manufacturer of the commercial inoculant. Three replicates of each treatment were included in the experiment and the treated plots were kept more than 50 m away from the untreated plots to avoid contamination with the microorganism. The pot experiment was a factorial design of two treatments with inoculant, with and without, and four N rates, corresponding to the application of 0, 0.4, 0.8, and 1.6 g pot^−1^, and four replicates per treatment. N rates aimed to provide each plant with the equivalent of 0, 40, 80, and 160 kg ha^−1^ of N, considering a planting density of 100,000 plants ha^−1^.

### 2.3. Preparation and Management of Field and Pot Experiments

In the field experiment, maize (hybrid DKC 6181, stay-green line, mid-season FAO 500) was sown on May 13 and 16, 2021 and 2022, respectively. Before sowing, the seedbed was prepared by plowing the soil to a depth of 0.25 m, followed by chiseling to level the ground. Subsequently, the plots were marked to receive pre-sowing fertilization. A compound NPK fertilizer (10:10:10; N:P_2_O_5_:K_2_O) was applied at a rate of 700 kg ha^−1^ of fertilizer (70, 70, and 70 kg ha^−1^ of N, P_2_O_5_ and K_2_O, respectively), which represents 70% of half the full rate of N recommended by the laboratory (200 kg ha^−1^ of N), following the manufacturer’s instructions for the best use of the commercial product Blue N. The fertilizer was then incorporated with a last pass of cultivator, which also finalized the preparation of the seedbed. Sowing was carried out with a precision seeder, which spaced the seeds at 0.70 m between rows and 0.14 m between rows (100,000 plants ha^−1^). Plots with and without inoculant were placed more than 50 m apart to eliminate the risk of bacterial contamination from the treated plots. At the phenological stage 14, four unfolded leaves [45], a herbicide treatment was carried out. The herbicide that was used contained isoxadifen-ethyl (22 g L^−1^) and tembotrione (44 g L^−1^) as active ingredients and was applied at a concentration of 0.5 L hL^−1^ (2 L ha^−1^).

The other half N rate (70 kg ha^−1^ of N, as ammonium nitrate, 20.5% N) was applied as a side-dressing when the plants were at phenological stage 16/17, six to seven unfolded leaves [45]. This application coincided with the application of the inoculant in the corresponding plots. The inoculant was applied at the concentration recommended by the manufacturer (333 g ha^−1^, diluted in 100 L of water) and applied with a knapsack sprayer, wetting the adaxial and abaxial sides of the leaves. Maize was sprinkled irrigated with a center pivot whenever the summer rains were not enough to keep the plant hydrated. According to estimates by the farmers’ association that supplies the water, approximately 3000 m^3^ ha^−1^ of water was used each year. No other farming operations were carried out during the growing season.

The pots (0.36 m in height and 0.17 m in average diameter) were filled with 10 kg of dried and sieved soil. In the initial phase, the fertilizers used in pre-planting were homogeneously mixed with the soil and then placed in the pots. As a pre-planting N fertilizer, half of the N rates mentioned in the experimental design were applied as ammonium nitrate (20.5% N). P was also applied, at a rate of 0.8 g (P_2_O_5_) pot^−1^ (as superphosphate, 18% P_2_O_5_) and potassium (K) at a rate of 0.8 g (K_2_O) pot^−1^ (as potassium chloride, 60% K_2_O). Many other macro- and micronutrients were applied using a fertilizer containing 10% MgO, 0.3% boron (B), 18.5% SO3, 0.3% copper (Cu), 2% iron (Fe), 1% manganese (Mn), 0.02% molybdenum (Mo), and 1.6% zinc (Zn) at the rate of 0.16 g pot^−1^ year^−1^. Sowings were carried out on May 24 and 27, 2021 and 2022, respectively. The hybrid DKC 6181 was also used in the pot experiment. Three seeds were placed in each pot. After germination, excess seedlings were removed, leaving only one plant per pot. In the following weeks, all the weeds that were germinating in the pots were promptly pulled out. In the phenological stage 16/17 [45], the pots corresponding to the inoculant treatment received the commercial product as a foliar spray at the dosage and concentration referred to the field, with each plant receiving the fraction corresponding to each of the 100,000 plants of a hectare. The application was carried out with a sprayer suitable for treating indoor plants, adequately wetting the adaxial and abaxial sides of the leaves. On that date, N was also applied as side-dressing, at an equivalent rate to that applied in pre-planting.

Pots were watered throughout the growing season as needed. Considering the variation in environmental variables during the growing season, the phenological state of the plants, and the differences in plant size induced by the fertilizer treatments, the pots received different amounts of water, but care was taken not to over-water or that the plants went through periods of drought stress. After sowing, the pots that received the inoculant were placed 50 m away from the pots that did not receive the inoculant. All pots were surrounded by a wooden plank structure to prevent solar radiation from falling directly on the sides of the pots and excessively increasing the temperature in the rooting zone. No further cropping practices were needed throughout the growing season until harvest.

### 2.4. Measurements During the Growing Season

Leaf greenness, an index of plant N nutritional status, was determined in the field and pot experiments using the portable Soil and Plant Analysis Development (SPAD)-502 Plus chlorophyll meter (Spectrum Technologies, Inc., Osaka, Japan). In the field, thirty readings for each measurement were taken from the middle of the blade of the youngest fully expanded leaves. In the pot experiment, five readings for each plant were taken also from the youngest fully expanded leaves.

### 2.5. Sample Collection and Preparation for Laboratory Analysis

At the beginning of the field trial, three composite soil samples (10 cores per sample) were taken from the 0–0.20 m layer to characterize the experimental plot. For the pot experiment, the soil was initially also taken in the 0–0.20 m layer in sufficient quantity for all pots. Then, three samples were taken for laboratory analysis by a random process. All soil samples were dried in an oven at 40 °C and sieved (2 mm mesh) before being submitted for laboratory analysis.

In the field experiment, the final harvests were carried out on September 12 and 19, 2021 and 2022, respectively, at phenological stage ~73 (early milk) [45]. For each plot, 10 plants were taken from two central rows of the plots. After cutting the plants close to the ground, the samples were immediately weighed fresh and chopped into thin sections. Then, random subsamples of approx. 1 kg of fresh matter were taken and placed in hermetically sealed plastic bags. In the laboratory, the subsamples were weighed fresh, oven-dried at 70 °C until constant weight, and weighed to determine the percentage of dry matter, data that allowed estimating the DMY in the plot and in the hectare. Thereafter, the samples were ground (1 mm mesh) and sent for elemental chemical analysis.

The plants in the potted experiment were harvested on 7 and 12 September, 2021 and 2022, respectively, at the phenological stage 87 (physiological maturity) [45]. Individual plants were oven-dried at 70 °C to constant mass and ground (1 mm mesh) before being sent for elemental analysis.

### 2.6. Soil and Plant Analysis

Soil samples were analyzed for their content on clay, silt, and sand by the Robinson pipette method. After that, they were also analyzed for pH (H_2_O and KCl) (soil: solution, 1:2.5), cation-exchange capacity (ammonium acetate, pH of 7.0), organic C (wet digestion, Walkley–Black method) and extractable P and K (Egner–Riehm method, ammonium lactate extract). These analytical procedures are fully described in Van Reeuwijk [46].

The N concentration in plant tissues was quantified using the Kjeldahl method. This procedure involves mineralizing the tissue sample with sulfuric acid (H_2_SO_4_) and a selenium-based catalyst. Following mineralization, the sample undergoes distillation with sodium hydroxide (NaOH), which converts the N into ammonia (NH_3_). The amount of ammonia is then determined by titration, which measures the NH_3_ carried in the steam stream [47].

### 2.7. Data Analysis

Data were tested for normality and variances homogeneity using the Shapiro–Wilk and Bartlett’s tests, respectively. The results of the field experiment were compared using a Student’s *t*-test to compare two means. A two-way ANOVA examined the results of the pot experiment. When the means of the N treatments displayed significant differences (*p* < 0.05), they were separated by Tukey HSD test (α = 0.05)

Apparent N fixation (ANF) was used as an index of the effectiveness of N fixation by the microorganism in both the field and pot experiments. ANF was determined by the difference between N recovered by plants that were and were not treated with the N-fixing microorganism separately for each rate of N applied to the soil: ANF = N recovered in inoculated plants—N recovered in untreated plants.

## 3. Results

### 3.1. Maize Growth Performance

**Figure 2:**
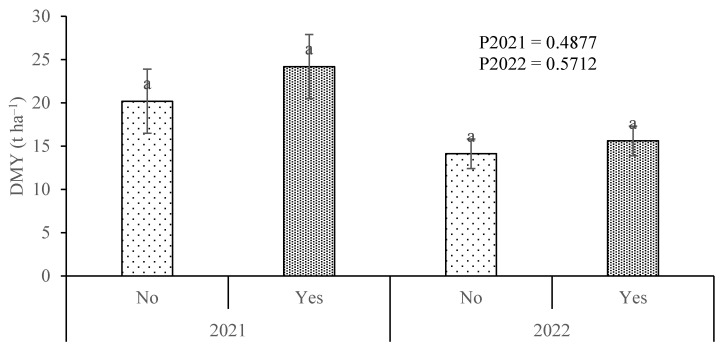
Maize dry matter yield (DMY) in the field experiments of 2021 and 2022 for the treatments without (No) and with (Yes, 333 g ha^−1^) inoculant. Within each year, means followed by the same letter are not significantly different according to Student’s *t*-test. The error bars represent the standard errors.

In the field experiment, no significant differences were observed between treatments in DMY, either in 2021 or 2022 (Figure 2). However, in 2021, the average yield of the plots that received the inoculant (24.2 t ha^−1^) was higher compared to those that did not receive it (20.2 t ha^−1^). In 2022, the average yields were closer, with 15.6 t ha^−1^ and 14.1 t ha^−1^ for the plots that received and did not receive the inoculant, respectively.

**Figure 3:**
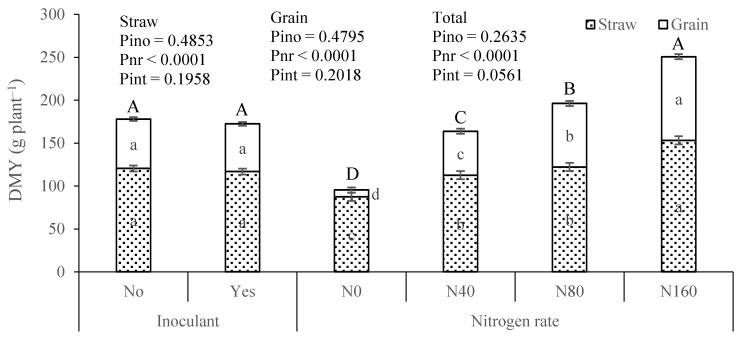
Dry matter yield (DMY) of maize straw, grain, and total in 2021 from the factorial experiment involving inoculant application [No and Yes (333 g ha^−1^)] × nitrogen rate [0 (N0), 40 (N40), 80 (N80), and 160 (N160) kg ha^−1^]. Pino, Pnr, and Pint represent the probability associated with inoculant, nitrogen rate, and their interaction, respectively. For each experimental factor, means followed by the same letter within (lowercase) and above (uppercase) the columns are not significantly different by the Tukey HSD test (α = 0.05). The error bars represent the standard errors.

The results from the pot experiment did not reveal significant differences in maize straw, grain, and total DMY due to the application of the inoculant (Figure 3). The average values for straw and grain were 120.4 and 57.6 g pot^−1^, and 116.8 and 55.6 g pot^−1^ for the treatments without and with inoculant, respectively. The application of mineral N, in turn, resulted in highly significant differences between treatments in straw, grain, and total DMY. The total DMY varied significantly across the different levels of applied N, with average values of 95.4, 163.7, 196.1, and 250.6 g pot^−1^ for the N0, N40, N80, and N160 treatments, respectively.

**Figure 4:**
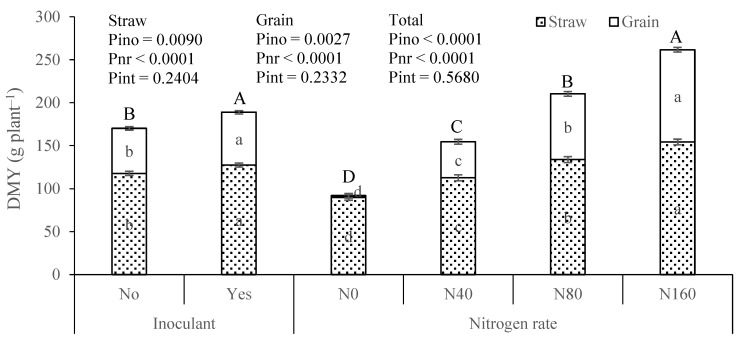
Dry matter yield (DMY) of maize straw, grain, and total in 2022 from the factorial experiment involving inoculant application [No and Yes (333 g ha^−1^)] × nitrogen rate [0 (N0), 40 (N40), 80 (N80), and 160 (N160) kg ha^−1^]. Pino, Pnr, and Pint represent the probability associated with inoculant, nitrogen rate, and their interaction, respectively. For each experimental factor, means followed by the same letter within (lowercase) and above (uppercase) the columns are not significantly different by the Tukey HSD test (α = 0.05). The error bars represent the standard errors.

In the 2022 pot experiment, significant differences were observed in maize straw, grain, and total DMY due to the application of the inoculant (Figure 4). The total DMY averaged 170.2 and 189.0 g pot^−1^ for the treatments without and with inoculant, respectively. The results for mineral N application followed a pattern very similar to that of 2021, with significant differences observed across each N dose. The average total DMY values were 91.8, 154.5, 210.3, and 261.7 g pot^−1^ for the N0, N40, N80, and N160 treatments, respectively.

### 3.2. Plant N Nutritional Status, N Recovery and Apparent N Fixation

**Figure 5:**
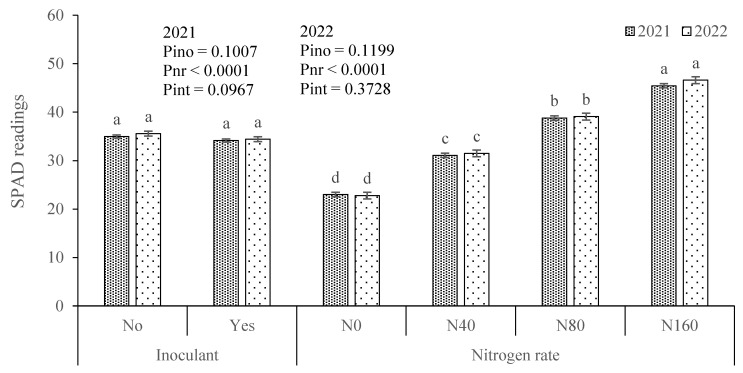
SPAD readings in maize leaves from the factorial experiment involving inoculant application [No and Yes (333 g ha^−1^)] × nitrogen rate [0 (N0), 40 (N40), 80 (N80), and 160 (N160) kg ha^−1^]. Pino, Pnr, and Pint represent the probability associated with inoculant, nitrogen rate, and their interaction, respectively. For each experimental factor and year, means followed by the same letter are not significantly different by the Tukey HSD test (α = 0.05). The error bars represent the standard errors.

SPAD readings did not exhibit significant differences between treatments for the inoculant factor (Figure 5), even in 2022 when differences in DMY were observed (Figure 4). The average values ranged between 34.2 and 35.5. Conversely, the application of mineral N to the soil had a pronounced effect on the greenness of the leaves, with significant differences in SPAD values across treatments in both years of this study. In 2021, the average SPAD values ranged from 23.0 in the N0 treatment to 45.5 in the N160 treatment. Similarly, in 2022, these values varied from 22.8 to 46.6 in the same respective treatments.

The N concentration in the whole plant at harvest in the field trial did not vary significantly with the application of the inoculant in either 2021 or 2022 (Table 2). The average values ranged between 9.4 and 10.3 g kg^−1^. The N recovered in the aboveground biomass, which is the product of N concentration and DMY, also did not differ significantly between treatments, although the average values were higher in the inoculant-treated plots. Consequently, the average ANF values were positive, at 58.7 and 14.5 kg ha^−1^, for the inoculant treatment in 2021 and 2022, respectively, and this represents 23.7% and 9.1% of the N recovered by maize.

The N concentration in the straw did not vary significantly with inoculant application in 2021 but was significantly lower in the inoculant-treated plots compared to the non-treated plots in 2022 (Table 3). The effect of soil N application on straw N concentration was generally higher in the N0 treatment and subsequently in the N160 treatment. It is worth noting that some plants in the N0 treatment did not produce grain, which hindered the remobilization of N from the straw. The N concentration in the grain decreased significantly with inoculant application in 2021 and increased in 2022, with a significant interaction observed with soil N application. The N application to the soil tended to be higher in the N0 treatment, where grain was produced in some plants and subsequently increased with the application of the highest N rate.

N recovery in the whole plant (grain + straw) did not vary significantly with inoculant application in 2021 but increased in 2022 compared to the non-treated control (Table 3). Soil N application had a marked effect on the amount of N recovered in the whole plant, with significant differences observed between N levels in both years. These N recovery results allowed for the estimation of average apparent N fixation values of −49.2 mg pot^−1^ in 2021 and 199.2 mg pot^−1^ in 2022, which represents −5.2% and 18.5% of the N recovered by maize, respectively. These quantities, distributed across the different soil N treatments, resulted in negative apparent N fixation values of −0.8, −88.2, −13.6, and −94.2 mg pot^−1^ in 2021, and overall positive values of 24.8, −14.3, 185.0, and 601.3 mg pot^−1^ in 2022, for the N0, N40, N80, and N160 treatments, respectively.

In this study, concentrations of other nutrients in plant tissues were also determined, including P, K, calcium (Ca), magnesium (Mg), boron (B), iron (Fe), manganese (Mn), copper (Cu), and zinc (Zn). Various soil properties were also assessed, such as organic C, pH, extractable P and K, base saturation, cation exchange capacity, and the micronutrients mentioned for plant tissue analysis. Overall, the variables analyzed showed limited sensitivity to the treatments and contributed minimally to the interpretation of the results and, therefore, are not presented.

## 4. Discussion

### 4.1. Dry Matter Yield Increased Significantly with Soil-Applied N and to a Much Lesser Extent with the Inoculant Application

In the field trial, no significant differences in DMY were observed between plots with and without the inoculant. However, the plots with the inoculant generally yielded higher average values, particularly in the 2021 trial. In the pot experiment, no significant differences existed between inoculated and non-inoculated plants in 2021. However, in 2022, the inoculated plants exhibited significantly higher DMY. In the pot experiment, the response to soil-applied N was striking in both years, with highly significant differences observed among the various N levels applied. N is a major ecological factor limiting plant productivity and, as it generally does not accumulate in soils in forms usable by plants, it is typically necessary to apply it annually to crops [48,49]. Thus, providing mineral N to the plants resulted in a strong productivity response (Figure 2 and Figure 3), which was also facilitated by pot cultivation where root expansion and access to soil N are more constrained [50,51]. However, in a study where plants showed a strong response to N fertilization, the effect of applying an inoculant containing a N-fixing microorganism was modest in comparative terms.

The use of N-fixing inoculants in agriculture is a well-established practice, with commercial development primarily focused on the use of specific rhizobial strains for legume inoculation [21,22,23]. Some attempts to use other N-fixing microorganisms (*Azotobacter* sp., *Azospirillum* sp., *Herbaspirillum* sp., *Bacillus* sp., etc.) have been made, though with much more limited success compared to the application of inoculants in nodulated legumes [32,39,52]. In maize cultivation, Alves et al. [34] applied the endophytic diazotroph *H. seropedicae* strain ZAE94. The authors observed that the amount of N found in the plants increased, indicating that biological N fixation had occurred. However, the practical significance of using these inoculants under field conditions remains uncertain and continues to be a topic of ongoing debate [32,34].

### 4.2. Indicators of N Nutritional Status Increased with Soil-Applied N but Did Not Change Significantly with the Inoculant Application

The plant greenness did not vary significantly with the inoculant application, but it increased significantly with each level of soil-applied N. SPAD values measure the light absorbed by leaf chlorophyll, with higher values indicating greater chlorophyll content [53]. Chlorophyll content, in turn, reflects the N content in the plant [48,49], and SPAD readings are commonly used as an indicator of the plant’s N nutritional status [54,55]. This result highlights the high effectiveness of supplying N to the plant through soil-applied N and the lower effectiveness of the inoculant used for the same purpose.

The total N concentration in the plant, as measured in the field trial, did not vary significantly with the inoculant application. In the pot experiment, the effects of inoculant application and N rate on N concentration in plant tissues were difficult to interpret due to the separate analysis of straw and grain. For instance, some plants in the N0 treatment did not produce grain, resulting in higher total N concentrations in straw compared to some treatments that received N. Substantial remobilization of photosynthates occurs in mature plants from the leaves to the developing reproductive organs, as these organs are prioritized sinks for these resources [56,57]. Regarding N, there is a noticeable reduction in leaf N concentration throughout the growing season as the nutrient is remobilized to the seeds, where it tends to accumulate in the form of protein [49,58]. In the N0-treated plants, N deficiency was so severe that the plants did not produce ears, leading to generally higher N concentrations in the straw compared to the fertilized treatments.

### 4.3. The Inoculant Application Was Inconsistent in N Fixation

When evaluating the N recovery in plants during the field trial, no significant differences were observed between treatments, although the mean values were higher in inoculated plants. In the 2022 pot experiment, plants treated with the inoculant showed significantly higher total N recovery (grain + straw) than untreated plants. However, the results from the mineral N treatments demonstrated a pronounced effect on the amount of N recovered in the whole plant, indicating that the additional N accessible to inoculated plants was relatively modest by comparison. On average, the field trial estimated N fixed through inoculant application at 57.7 and 14.5 kg ha^−1^ in 2021 and 2022, respectively, while in the pot experiment, the values were −49.2 and 199.2 mg pot^−1^ in 2021 and 2022, respectively. These N fixation values resulting from inoculant application can be considered modest, especially compared to the increase in N recovery with soil-applied N. These findings align with the results of other recently published studies using the same inoculant [51,59].

The capacity for biological N fixation by N-fixing microorganisms is highly dependent on the systems in which they are integrated. High N fixation capacities can be achieved in symbiotic systems associated with nodulated legumes. Microorganisms invade the host tissues after the plant secretes secondary metabolites called flavonoids, which are recognized by the bacteria, prompting the release of lipochitooligosaccharides known as nodulation factors, which are then recognized by the host plant [60]. Once inside the plant tissues, the microorganisms transform into bacteroids through the synthesis of leghemoglobin, which regulates oxygen flow within the nodules. This process, combined with the supply of photosynthates via the phloem, ensures a high N fixation capacity, which under certain conditions can exceed 400 kg ha^−1^ year^−1^ [21,22,23].

In other N-fixing systems, such as the relationship between the aquatic fern Azolla filiculoides and the cyanobacterium *Anabaena azollae*, N fixation can exceed 100 kg ha^−1^ year^−1^ [21,23,24,61]. This fern is often used as an intercrop with rice to reduce the need for N fertilization [24,25]. Numerous cavities on the fern’s leaf surfaces house the cyanobacterium, protecting it from predation and facilitating access to exudates, which allows for high levels of N fixation to be achieved [24,61,62].

It is also well documented that some tropical grasses, such as sugarcane can establish endophytic associations with N-fixing microorganisms like *Gluconoacetobacter diazotrophicus* and *Azospirillum brasilense*, which can satisfy more than half of the plant’s N needs [26,27,62]. *H. seropedicae* is also considered a true endophytic diazotroph predominantly associated with tropical grasses, capable of invading the roots, stems, and leaves of the host plant, particularly the apoplastic compartments, although the quantities of N fixed by this bacterium are less well documented [31,32,33,34].

In all high-capacity N-fixing systems, highly specific symbiotic relationships are established between symbionts, resulting from millions of years of coevolution [23,61]. However, this does not preclude numerous free-living N fixers, which do not depend on a host, from accessing atmospheric N. These microorganisms can inhabit water as autotrophs, live on leaf surfaces, or exist in the soil as heterotrophs. However, excluding the photoautotrophs, N fixation capacities are generally reduced due to the lack of protection for nitrogenase from excess oxygen and substrate limitations [21,23,63].

The Blue N inoculant was isolated from *Glomus iranicum* var. *tenuihypharum* spores [35]. It is known that other bacteria of the genus *Methylobacterium* (e.g., *M. nodulans* and *M. radiotolerans*) are capable of fixing N in interaction with plants by forming nodules on legume roots [40]. However, the Blue N inoculant, which contains *M. symbioticum*, was developed for foliar application, allowing it to thrive on the phyllosphere of cultivated plants. Some N-fixing microorganisms can live on the phyllosphere of higher plants, where they have access to various plant-released products such as methanol, which they use as a C source, as well as soluble carbohydrates, amino acids, organic acids, and many other compounds. This access may enhance their N fixation capacity compared to free-living microorganisms [39,40,41].

According to studies conducted by the team that developed the commercial product, applying the inoculant to maize and strawberries resulted in a 50% and 25% reduction in the required N, respectively, accompanied by an increase in productivity compared to treatments that received the same amount of soil-applied N but without the inoculant application [38]. However, the Blue N inoculant is generally recommended for all cultivated species, and as is well known, each species harbors a diverse microbiome, with different species competing for photosynthates and space [39,64,65]. Furthermore, the phyllosphere is subject to variable environmental conditions, which can be either hostile or favorable to specific microorganisms [39,64,65]. Although studies quantifying these effects are still lacking [39,42], this environmental variability may account for the considerably different outcomes observed in the four trials reported in this study. This variability makes it highly unlikely that the product could be universally effective, as high N fixation capacities typically result from highly specific relationships between N-fixing microorganisms and their host plants [21,22,23]. Consequently, the variation in results across the four trials underscores the urgent need for additional research to better establish the conditions under which a commercial inoculant can provide more consistent and predictable outcomes for farmers, depending on plant species and cultivation conditions.

In the pot experiment, a significant interaction was observed between the inoculant application and the soil N application. This means that the N recovered in the inoculant-treated plants depended on the level of mineral N applied and vice versa. Consequently, the 2021 results showed that inoculant application tended to be more negative at higher levels of soil-applied N; meanwhile in 2022, the results tended to be more positive. The commercial product information suggests that this inoculant is more effective when plants have a moderate N nutritional status than when N levels are very low [38,66]. It is believed that plants with a moderately favorable nutritional status release more substrates that microorganisms can utilize. The results from the second year seem to support this hypothesis, although the first-year results tend to complicate the overall interpretation. On the other hand, although the environmental variables reported for this study, namely average air temperature and precipitation (Figure 1), do not clearly establish the cause of the differences observed in the various trials, and considering that the soil was the same in both years of this study, it is evident that the efficacy of the inoculant is sensitive to the conditions of application. Therefore, these conditions must be better understood to ensure greater reliability in the efficacy of the results when the inoculant is used by farmers.

## 5. Conclusions

The commercial inoculant Blue N demonstrated limited consistency in fixing N when applied to maize crops in field and pot experiments. The limitations of the inoculant were more evident in the pot experiment, where plants showed a strong response to various N rates applied to the soil. To date, high N fixation capacity has been observed in biological systems with a high specificity between the N-fixing microorganism and the host plant, resulting from a coevolutionary process spanning millions of years. To ensure that the use of this commercial inoculant is more effective and yields more reliable results for farmers, further studies are necessary to optimize application conditions. We should not overlook that in a global context, where access to industrial N remains linked to very high energy costs and the use of synthetic fertilizers can have significant negative environmental impacts, alternative or supplementary fertilization methods are particularly important on the path toward more sustainable agriculture.

## Figures and Tables

**Table 1:** Selected soil properties (average ± standard deviation, *n* = 3) determined from composite soil samples taken at 0–0.20 m depth at the beginning of the experiments.

Soil Properties	Field Trial	Pot Experiment
^1^ Organic carbon (g kg^−1^)	14.1 ± 0.61	9.1 ± 1.26
^2^ pH (H_2_O)	5.9 ± 0.31	6.5 ± 0.20
^3^ Extract. phosphorus (mg kg^−1^, P_2_O_5_)	44.0 ± 8.96	67.2 ± 13.78
^3^ Extract. potassium (mg kg^−1^, K_2_O)	103.7 ± 11.48	81.2 ± 7.71
^4^ Exchang. calcium (cmol_c_ kg^−1^)	13.7 ± 0.92	9.8 ± 1.21
^4^ Exchang. magnesium (cmol_c_ kg^−1^)	4.9 ± 0.44	3.5 ± 0.16
^4^ Exchang. potassium (cmol_c_ kg^−1^)	1.1 ± 0.16	0.3 ± 0.03
^4^ Exchang. sodium (cmol_c_ kg^−1^)	1.5 ± 0.12	0.4 ± 0.04
^5^ Exchang. acidity (cmol_c_ kg^−1^)	0.1 ± 0.00	0.1 ± 0.02
^6^ CEC (cmol_c_ kg^−1^)	21.3 ± 1.08	14.1 ± 1.34
^7^ Sand	562.1 ± 28.55	544.1 ± 24.25
^7^ Silt	245.3 ± 22.89	206.9 ± 20.88
^7^ Clay	192.7 ± 50.77	249.0 ± 43.31
^8^ Texture	Sandy loam	Sandy clay loam

^1^ Wet digestion (Walkley–Black); ^2^ potentiometry; ^3^ ammonium lactate; ^4^ ammonium acetate; ^5^ potassium chloride; ^6^ cation exchange capacity; ^7^ Robinson pipette method; ^8^ USDA (The United States Department of Agriculture).

**Table 2:** Plant nitrogen concentration (PNC), N recovery, and apparent N fixation (ANF) in the field experiments of 2021 and 2022 for the treatments without (No) and with (Yes, 333 g ha^−1^) inoculant.

	PNC (g kg^−1^)		N Recovery (kg ha^−1^)		ANF (kg ha^−1^)	
	2021	2022	2021	2022	2021	2022
No	9.4 a	10.3 a	188.8 a	144.6 a		
Yes	10.2 a	10.1 a	247.5 a	159.1 a	58.7	14.5
Prob.	0.0503	0.7226	0.3008	0.6385		

In columns, means followed by the same letter are not significantly different by the Student’s *t*-test. ANF = N recovery in inoculated plants − N recovery in untreated plants.

**Table 3:** Straw nitrogen concentration (SNC), grain N concentration (GNC), N recovery in the whole plant (grain + straw), and apparent N fixation (ANF) in 2021 and 2022 from a factorial experiment involving inoculant application [No and Yes (333 g ha^−1^)] × nitrogen rate [0 (N0), 40 (N40), 80 (N80), and 160 (N160) kg ha^−1^]. Pino, Pnr, and Pint represent the probability associated with inoculant, nitrogen rate, and their interaction, respectively.

	SNC (g kg^−1^)		GNC (g kg^−1^)		N Recovery (mg pot^−1^)		ANF (mg pot^−1^)	
	2021	2022	2021	2022	2021	2022	2021	2022
Inoculant								
No	4.2 a	3.2 a	9.7 a	10.0 b	1002.4 a	880.4 b		
Yes	4.1 a	3.0 b	8.4 b	12.0 a	953.1 a	1079.6 a	−49.2	199.2
Nitrogen rate								
N0	4.4 a	4.1 a	13.6 a	----	475.6 d	369.9 d	−0.8	24.8
N40	3.9 b	2.8 bc	8.3 bc	10.7 b	859.7 c	685.3 c	−88.2	−14.3
N80	3.6 c	2.5 c	7.7 c	9.7 c	1021.2 b	1074.1 b	−13.6	185.0
N120	4.6 a	2.9 b	8.9 b	12.4 a	1565.3 a	1790.8 a	−94.2	601.3
Pino	0.0811	0.0031	<0.0001	<0.0001	0.1968	0.0003		
Pnr	<0.0001	<0.0001	<0.0001	<0.0001	<0.0001	<0.0001		
Pint	<0.0001	0.0221	<0.0001	0.0001	0.6446	0.0004		

In columns, means followed by the same letter are not significantly different by the Tukey HSD test (α = 0.05). ANF = N recovery in inoculated plants − N recovery in untreated plants.

## Data Availability

The data presented in this study are available on request from the corresponding author.
